# Bambara groundnut (*Vigna subterranea* (L.) Verdc.) flour: A functional ingredient to favour the use of an unexploited sustainable protein source

**DOI:** 10.1371/journal.pone.0205776

**Published:** 2018-10-15

**Authors:** Juliet Mubaiwa, Vincenzo Fogliano, Cathrine Chidewe, Anita R. Linnemann

**Affiliations:** 1 Department of Food Science and Technology, Chinhoyi University of Technology, Chinhoyi, Zimbabwe; 2 Food Quality and Design, Wageningen, The Netherlands; 3 Department of Biochemistry, University of Zimbabwe, Mount Pleasant, Harare, Zimbabwe; College of Agricultural Sciences, UNITED STATES

## Abstract

Variability in dehulling efficiency, colour, chemical composition and selected functional properties of raw and pre-treated bambara groundnut (*Vigna subterranea* (L.) Verdc.) (BG) flour from red and black-eye varieties were studied. Functional properties were water and oil absorption, gelation, pasting, emulsification and foaming capacity. Pre-treatment of seeds (i.e. soaking, roasting and combined soaking and roasting) improved dehulling efficiency of BG varieties. Protein content of flour ranged from 15.6–19.6%, starch from 47.8–52.0% and sucrose from 1.9–5%. An improvement was observed for protein and ash content of pre-treated flour compared to raw flour. Heat treatments increased onset gelatinization temperature of flour. Black-eye BG flours that had higher starch content, also had better gelation capacity than red BG flours. All pre-treatment methods decreased flour emulsification capacity and stability. Dry-roasting caused a greater decline than other methods, whereas soaking had little effect on emulsion stability. Further, soaking increased foaming capacity, whilst a decline was observed in roasted flour. All pre-treatment methods increased oil absorption capacity of both BG flour varieties. Overall, soaked and combined soaked and roasted flour is recommended for BG flour to be incorporated in food products.

## Introduction

Impact analyses in sub-Saharan Africa (SSA) indicated that indigenous legume crops can reduce vulnerability of rural households to food and nutrition insecurity [1]. One such legume is bambara groundnut (BG) (*Vigna subterranea* (L.) Verdc.), which features in subsistence farming systems throughout semi-arid regions of SSA [2]. Bambara groundnut is an important source of affordable protein in the diets especially in regions where animal protein is comparatively expensive. The crop is richer in essential amino acids than other legumes and has a higher protein score (80%) than soya bean (74%) and cowpea (64%) [3]. This means that bambara groundnut has relatively more protein available for human metabolism than the other common legumes in Africa [4]. However, like other legumes, BG lacks sulphur containing amino acids [5, 6]. Thus, blending bambara groundnut with a staple food, such as maize, which contains higher levels of cysteine and methionine, is a recommended nutritional strategy [7].

Although BG is a crop with great nutritional and agronomic potential, it remains underutilized. Drawbacks to use BG and other legumes are the hard-to-cook (HTC) and hard-to-mill (HTM) phenomena [8]. The HTC phenomenon in legumes is characterised by an extended cooking time required to ensure adequate softening prior to consumption. In rural areas where BG is of significance, the predominant energy source for cooking is firewood. Due to limited firewood supply and the long time required for boiling, consumption of BG is hampered [9], thus warranting the need for alternative processing methods [10]. A recent survey performed by our group Mubaiwa, Fogliano [11] revealed that soaking and or roasting are applied by some households as pre-treatment methods prior to milling of BG seeds into flour in an effort to circumvent the HTC problem. In previous studies, soaking [12], germination [13] and roasting are also employed to bypass the HTM phenomenon.

BG flour has several applications in households to prepare porridge, soups and baked products. However, successful performance of BG flour as a food ingredient depends on the functional characteristics and sensory qualities it imparts to products. Functional properties are intrinsic physico-chemical characteristics that affect behaviour of foods during pre-treatment and storage, e.g. solubility, foamability, gelation and emulsification.

A study by Onimawo, Momoh [14] reported low water binding capacities of raw BG flour as compared to cowpea and soya bean flours. In addition, low foaming properties and stability were also reported, suggesting unsuitability of BG flour for cakes and bread. In a study by Yagoub and Abdalla [15], combined soaking and cooking of BG seeds as well as roasting resulted in the least solubility of nitrogen, whereas soaking led to better solubility. Further, according Olapade and Adetuyi [16], foaming capacity was highest for a cold-water soaked flour (22%) while 13% was reported for roasted flour. Low foamability of roasted flour was attributed to ordered globular molecules due to the high temperature (190°C) employed during roasting [16].

To improve the use of BG flour as a food ingredient, the functional properties and the characteristics imparted to food stuffs need to be assessed. Eventually, consumption of BG can be stimulated by processing seeds into a flour, easing the utilisation and providing more diversity in local diets. Improving the use of BG would mean an increase in food and nutrition security in SSA, while complying with local food preferences and habits. The aim of the paper is to investigate processing effects on chemical composition and functional properties of BG flour from different varieties. Such knowledge allows to decide if varietal differences should be accounted for and which pre-treatment results in the best flour for a particular application.

## Materials and methods

### Sample characteristics and flour production

**Fig 1:**
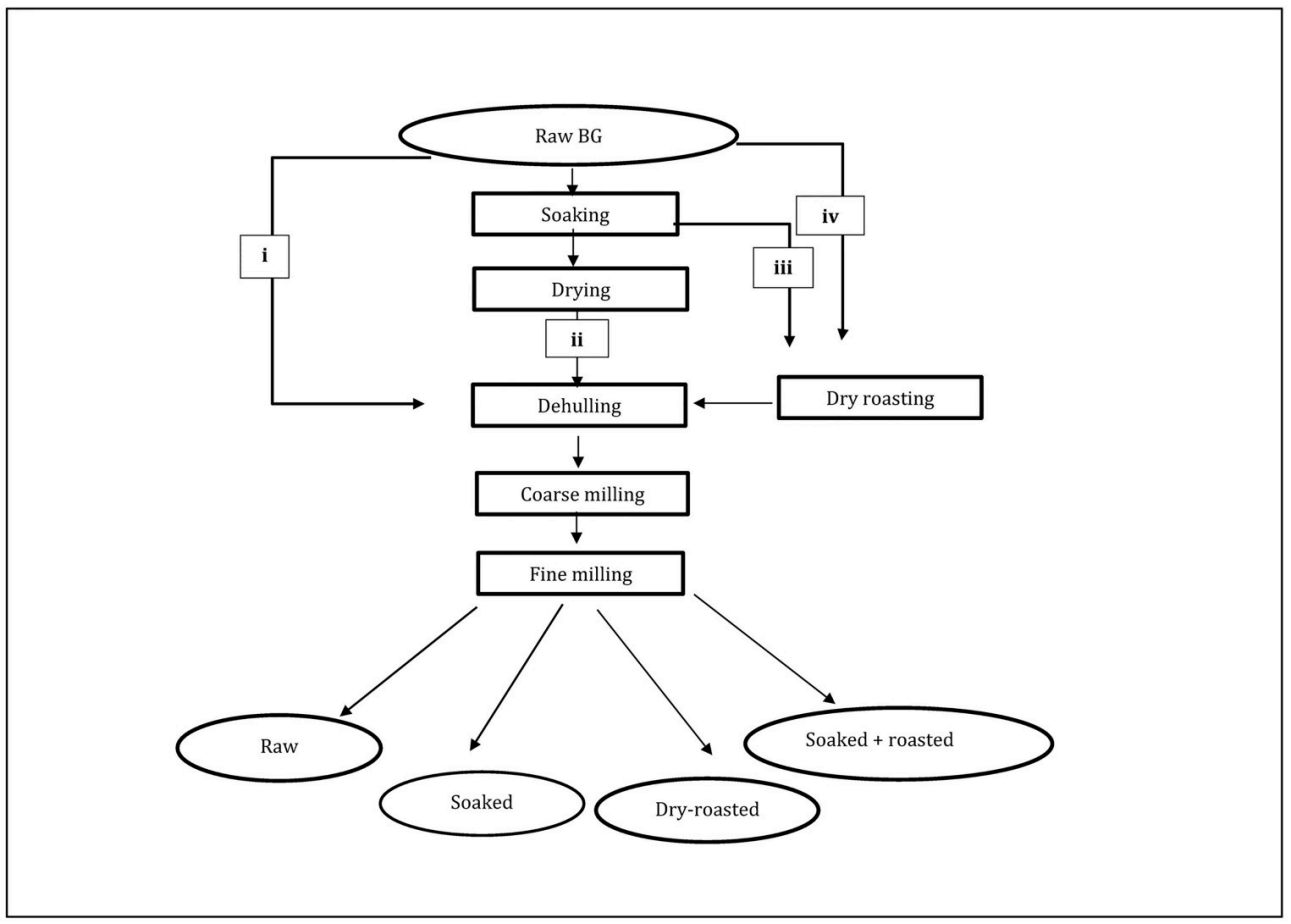
Pre-treatments of bambara groundnut before coarse and fine milling to flour.

A red and a black-eye seeded variety of BG were procured in Zimbabwe from the Director of Research and Specialist Services (DRSS) and Dee Spice Private Company, respectively. The varieties were grown in the 2016–2017 farming season, harvested and stored at ambient temperatures. Batches of 2 kg seeds each underwent different pre-treatments: i) no pre-treatment (raw), ii) soaking, iii) soaking followed by dry roasting and iv) dry roasting as outlined in Fig 1. Soaked seeds were prepared by immersing in excess deionised water for 24 h at 25°C followed by drying in an oven for 48 h at 50°C. Dry-roasted seeds were prepared by roasting in a Hot top Coffee bean roaster (KN-8828-2K, Pullman Espresso Accessories, Australia), following programme 6 for 10 min to standardize the roasting process. In programme 6, subsequent temperature ranges were maintained for 1 min (73–70°C, 70–80°C, 80–90°C, 90–106°C, 106–120°C, 120–132°C, 132–145°C, 145–162°C, 162–167°C, 167–179°C), followed by 5 min cooling. For the combined soaking and roasting treatment, seeds were soaked as in ii) and roasted as in iii). Next, all samples were mechanically dehulled using a SATAKE-TMO-5C dehuller. Dehulling efficiency (DE) was determined according to Enwere and Hung [12]. Next, pre-treated and raw seeds were coarse milled using a Rotormill (Condux Werk, Germany) to a flour that passed through a 1.5 mm sieve. Thereafter, about 25 g of coarse flour was fine milled using an IKA blender (Model A 11B S000, Germany) for 30 s before sieving through a 180-μm sieve to obtain flour. Milling efficiency (%) was determined by calculating the yield before and after fine milling and sieving.

#### Gravimetric properties of raw and pre-treated seeds

Bulk density was determined by filling a 1000 ml container with kernels from a height of about 15 cm, striking the top level and then weighing the contents [17]. The seed true density was by the water displacement method according to Karababa [18]. The porosity of bulk seed was computed from the values of true density and bulk density according to Mohsenin [19].

#### Image analysis

Image analysis of raw and pre-treated bambara groundnut seeds was by X-ray computer Tomography (XRT / CT), allowing non-invasive 3D imaging of internal structures using GE/Phoenix v[tome]x m X-ray microfocus and nanofocus CT scanner.

#### Particle size distribution of flour

Flour was dispersed in ethanol and sonicated according to Kaptso, Njintang [20] before measuring particle size distribution with a Malvern MSS laser diffraction system (Malvern Instruments Ltd, Malvern, England). The Fraunhofer diffraction model, assuming a standardized spherical shape, was used for the analyses [21].

#### Colour measurements of raw and pre-treated flour

Colour was measured using Hunterlab colourflex EZ. Differences were determined by calculating chroma, hue and ΔE*.

### Chemical composition of flour

Dry matter, fat and ash content of BG flour were determined in duplicate according to methods described by Association of Official Analytical Chemists [22]. Analysis of protein content was according to the Dumas method using a Flash EA 1112 protein analyser (Thermo Scientific, Netherlands). Protein content was obtained using 5.7 as conversion factor [23]. Total starch was measured using AACC-76-13.01/AOAC 996.11 method (Megazyme K-TSTA-50A/K-TSTA) [24].

#### Quantification of carbohydrate content in flour

Flour (2.5 g) was dispersed in 25 ml Milli-Q/ethanol mixture (50–50% (v/v)) and centrifuged (10 min, 3000 rpm). The supernatant was filtered (RC HPLC filter, RC Minisart Satorius, Germany) and centrifuged (15 min, 14,000 rpm). A calibration curve was made for D-fructose (Merck) and D-sucrose (Sigma Aldrich). Quantification of carbohydrates (sucrose and fructose) was by HPLC using an Evaporative Light Scattering Detector (ELSD) [25] and the Alltech prevail Carbohydrates ES 5u 250*4.60 mm column. Flow rate was 0.8 ml/min for 35 min using 100% acetonitrile and deionised water as eluents.

### Determination of the functional properties of flour

#### Thermal properties

Thermal properties of flour were analysed using a Differential Scanning Calorimeter (DSC) equipped with a thermal analysis data station (Perkin-Elmer Corp Norwalk. USA) according to Evageliou, Richardson [26]. Flour (10–15 mg) was dispersed in distilled water (1:3 w/v) in hermetically sealed stainless steel capsules and incubated for 4 h to equilibrate moisture. Sample and reference pans (balanced to within ± 0.5 mg) were loaded at ambient temperature, cooled to 10°C, and held for 2 min before scanning to 120°C. Samples were scanned at a heating rate of 0.5°C/min using a Seteram microcalorimeter. Temperatures of characteristic transitions and enthalpy (ΔH) of transitions were recorded.

#### Least gelation concentration

Least gelation concentration (LGC) was determined in triplicate according to [27].

#### Pasting properties

Pasting properties were determined according to Afolabi [28] using a Rapid Visco Analyser (RVA 4500 Perten instruments, SIN 214 31208-45A, Australia). A suspension of 3 g of flour in 25 ml distilled water was subjected to controlled heating and cooling cycle under constant shear. During this cycle samples were held at 50°C for a minute, heated from 50 to 95°C at 6°C/min, held at 95°C for 5 min, cooled to 50°C at 6°C/min, and held at 50°C for 5 min. The pasting temperature, peak viscosity, trough viscosity, breakdown viscosity, final viscosity and setback viscosity (SB) values were obtained from the RVA curves and viscosity was expressed as centipoise units (Cp).

#### Foaming capacity and stability

Foaming capacity (FC) and stability (FS) were determined in triplicate as whip-ability of flour dispersed in water according to Coffmann and Garciaj [27]. Flour (5 g) was dispersed in 100 ml of deionised water and blended for 5 min using a Waring Laboratory blender at high speed. The blended mixture was poured into a 250 ml graduated cylinder and total height and height of the emulsion layer were measured immediately and after 1, 2, 4 and 6 h of room temperature incubation. FC and FS were calculated according to Eltayeb, Ali [29].

#### Emulsification capacity and emulsion stability

Emulsification capacity (EC) and emulsion stability (ES) were determined according to Chaparro Acuña, Gil González [30] with some modifications. Flour samples (0.4 g), 20 ml of maize oil (0.9 ml/g) and 20 ml water (1.0 ml/g) (resulting in a 1% (w/v) mixture) were homogenized for 1 min using a Waring blender at low speed. Thereafter, the mixture was centrifuged for 10 min at 1600 rpm and the height of the total content (TC) and the height of the emulsion layer (ELH) were measured. EC was determined by heating the sample at 80°C for 30 min.

#### Water and oil holding capacity

Water and oil holding capacity (WAC and OAC) were determined using the method of Diedericks and Jideani [31].

### Statistical analysis

Results were analysed and expressed as mean values with standard deviations. Two-way ANOVA statistical analysis was performed to determine significant differences amongst means. When significant differences (p < 0.05) were found, results were compared using a post-hoc test (Tukey). All statistical analyses were run using SPSS v 23.0 software.

## Results and discussion

### Effect of variety and pre-treatment on milling properties, colour and chemical composition of flour

#### Gravimetric and milling properties of raw and pre-treated seeds

Table 1 shows the variation of gravimetric and milling properties of raw pre-treated BG seeds. All pre-treatment methods decreased both the bulk and true density. This can be attributed to a higher rate of increase in seed volume than weight [18, 32]. Porosity of seeds ranged between 41–51%; in both varieties, soaking decreased seed porosity significantly agreeing with Sreerama, Sashikala [33], whereas insignificant porosity variation was found in dry roasting and combined soaking and roasting treatments.

**Table 1:** Gravimetric and milling properties of bambara groundnut flour made from a red and a black-eye variety.

Variety	Pre-treatment method	Bulk density	True density	Porosity (ɛ)	Dehulling	Milling	D_10_	D_50_	D_90_
		(g/cm^3^)		(%)	Efficiency (%)		Particle size diameter (μm)		
**Red**	**Raw**	0.63 ± 0.01^d^	1.23 ± 0.16^ab^	48.5 ± 6.1^a^	< 15%	66.0 ± 2.8^a^	10.4 ± 0.3^c^	36.3 ± 1.3^ab^	134.2 ± 14.1^a^
	**Soaked**	0.58 ± 0.02^bc^	1.04 ± 0.20 ^ab^	42.4 ± 13.2 ^a^	84.2 ± 1.9	73.2 ± 4.3^b^	7.1 ± 0.3^a^	30.5 ± 1.4^a^	129.1 ± 8.9 ^a^
	**Dry roasted**	0.58 ± 0.02^ab^	1.21 ± 0.20 ^ab^	51.1 ± 10.5 ^a^	83.8 ± 2.5	72.1 ± 2.3^ab^	9.7 ± 0.6^bc^	43.3 ± 2.6^bcd^	146.9± 23.1^a^
	**Soaked + roasted**	0.56 ± 0.01^ab^	1.05 ± 0.17 ^ab^	45.6 ± 8.8 ^a^	83.1 ± 4.1	74.3 ± 2.9^b^	10.6 ± 1.4^c^	48.6 ± 8.6^cd^	174.1± 51.8 ^a^
**Black-eye**	**Raw**	0.65 ± 0.02^d^	1.36 ± 0.16^b^	51.6 ± 5.0 ^a^	< 15%	65.9 ± 4.2^a^	8.5± 0.2 ^ab^	31.8 ± 0.8^a^	125.0± 1.8 ^a^
	**Soaked**	0.54 ± 0.01^a^	0.92 ± 0.02^a^	41.4 ± 2.6 ^a^	88.0 ± 6.0	72.3 ± 2.6^ab^	10.9 ± 0.6^c^	39.2 ± 2.6^abc^	152.3± 29.3 ^a^
	**Dry roasted**	0.62 ± 0.01^cd^	1.16 ± 0.02 ^ab^	46.7 ± 1.6 ^a^	85.7 ± 3.6	70.5 ± 0.8^ab^	9.8 ± 1.6^bc^	44.3 ± 11.1^bcd^	164.3± 39.5 ^a^
	**Soaked + roasted**	0.57 ± 0.01^ab^	1.19 ± 0.12 ^ab^	51.4 ± 5.2 ^a^	85.4 ± 4.0	72.3 ± 1.8^ab^	10.8 ± 1.2^c^	51.3 ± 5.4^cd^	152.3± 30.7 ^a^

Different superscript letters (a, b, c and d) in a column indicate means that are significantly different

According to Malik and Saini [32], a decrease in moisture content of seeds should be correlated to the decline in porosity as shown by the decline in moisture in all pre-treated seeds. However, an exception was in dry roasted red BG. Data of porosity are important to determine size reduction properties and the resistance to airflow during aeration and drying procedures such that a low porosity means a low heat transfer [17]. The significance of the observed results on porosity is that the raw and pre-treated seeds will differ in size reduction properties, which will affect homogeneity of particle size and the functional properties of flour.

**Fig 2:**
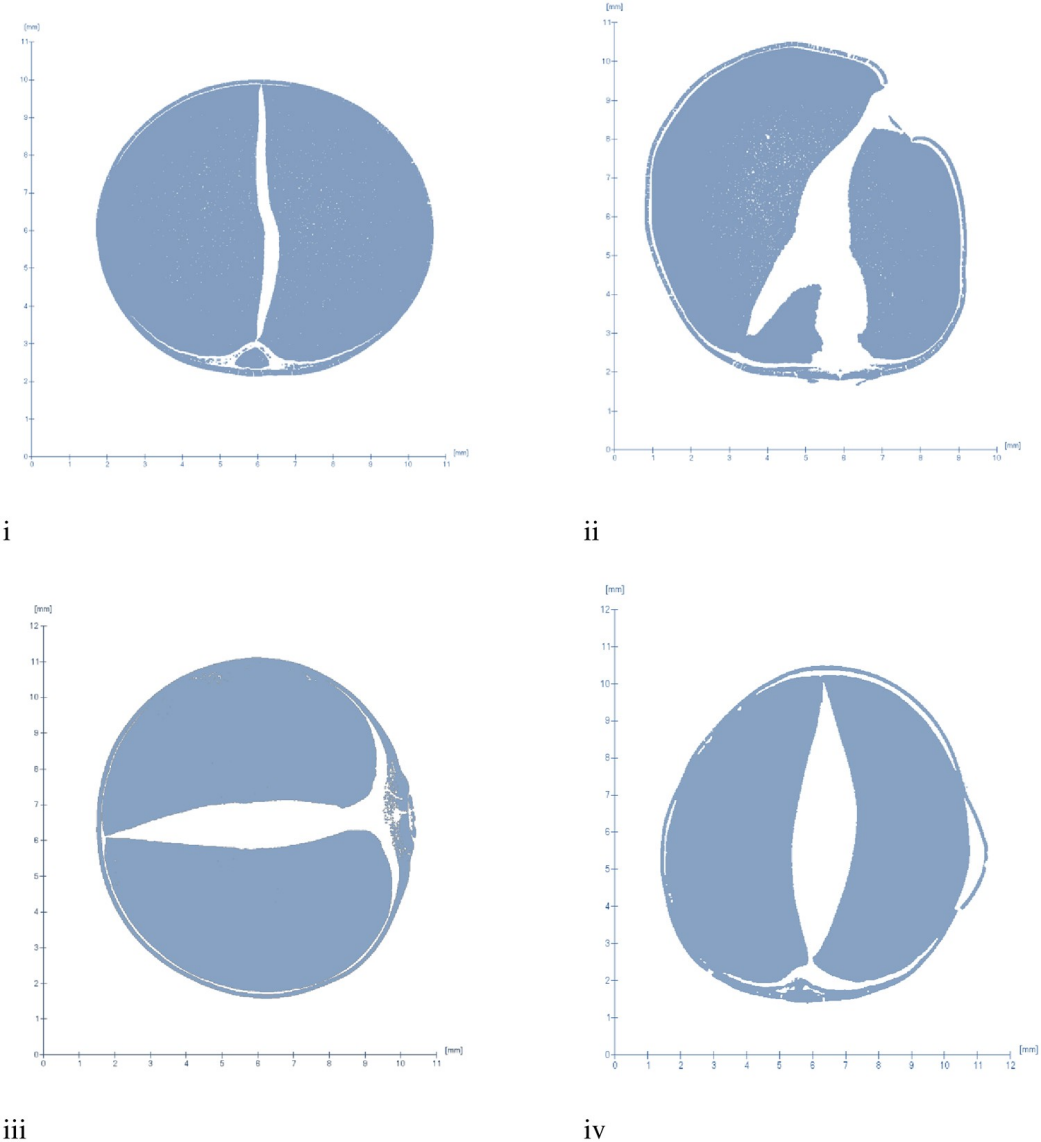
Image analysis of raw and pre-treated black-eye seeded bambara groundnut (i) raw, (ii) soaked, (iii) dry roasted, and (iv) soaked and roasted.

Raw seeds are very difficult to mill (HTM properties) as shown by a DE (%) of less than 15%, which increased to 83–88% when a dehulling pre-treatment was adopted. Fig 2 shows the images of raw and treated seeds, which display the differences in cotyledon and seed coat attachment. The low DE (%) of raw seeds is because the seed coat is strongly attached to the cotyledon, which makes removal difficult without pre-treatment [16]. However, treatment causes important changes. An insignificant effect of variety and pre-treatment (p > 0.05) on DE (%) found suggests that all pre-treatment methods were equally useful in managing the HTM characteristic. The positive effect of soaking on DE (%) in the current study agrees with the 81–88% reported by Enwere and Hung [12].

**Fig 3:**
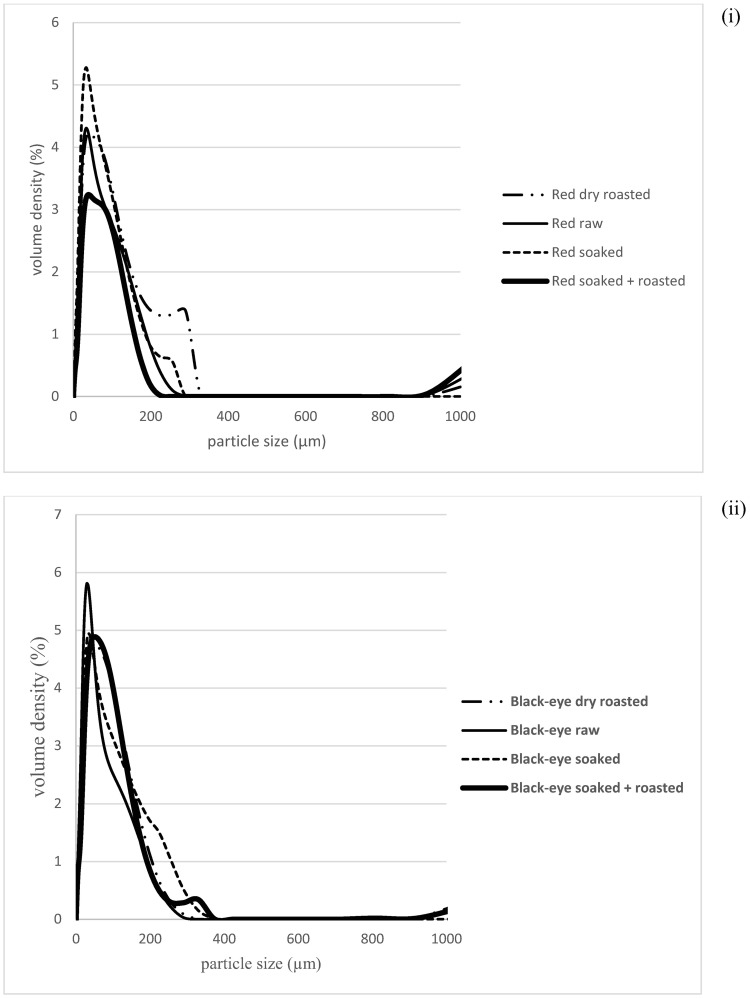
Particle size distribution of raw and pre-treated bambara groundnut flour from (i) a red and (ii) a black-eye variety.

Milling efficiency of raw coarse milled seeds increased after pre-treatment of seeds from 65% to 70–75% showing little distinction between varieties and methods, implying the applicability of all pre-treatments in circumventing HTM properties. Fig 3 shows the variation in particle size distribution of raw and pre-treated flour whereby a bi-modal distribution was evident in some red variety flours, agreeing with [34]. From the distribution curve, parameters d_10_, d_50_ and d_90_, which represent the diameter of 10, 50 and 90% of the population of the particles, are represented in Table 1. Ten percent of all samples had a diameter between 7.1–11 μm, 50% between 30–51 μm and 90% between 125–175 μm. As there were insignificant differences in 90% of the particle size, we expect a negligible effect of particle size on the characteristics imparted to the food products.

#### Colour of raw and pre-treated flour

Table 2 also shows ΔE*_ab_, chroma and hue values of flour made from red and black-eye varieties using different pre-treatment methods. Colour changes during roasting are ascribed to Maillard reactions occurring during roasting [35], influencing ΔE*_ab_, chroma and hue values of flour. The ΔE*_ab_ specifies differences between colour of control sample (raw) and pre-treated flour [36]. The ΔE*ab (0.14) of black-eye seeded raw and soaked flour shows that differences between flour were not perceptible by human eye. However, the ΔE*ab (1.2) of red seeded raw and soaked flour shows that differences between flour were perceptible through close observation. Exclusive roasting, combined soaking, and roasting processes are shown to have an effect on the colour of flour in both varieties such that colour differences from raw flour were perceptible at a glance. Even though soaking prior to roasting reduced ΔE*ab of flour of both varieties, differences in colour between exclusively roasted and combined soaked and roasted flour were still easily perceptible (ΔE*ab, 4.2–4.7).

**Table 2:** Colour of bambara groundnut flour made from red and black-eye varieties.

Variety	Pre-treatment method	Chroma (C*)	Hue (h°)	^a^ΔE*ab
**Red**	**Raw**	10.4^a^	1.51^e^	control
	**Soaked**	11.5^a^	1.55^f^	1.2
	**Dry roasted**	21.1^e^	1.39^b^	12.6
	**Soaked + roasted**	17.8^c^	1.45^d^	8.3
**Black-eye**	**Raw**	12.6^b^	1.57^g^	control
	**Soaked**	12.7^b^	1.56^g^	0.14
	**Dry roasted**	22.8^f^	1.37^a^	13.3
	**Soaked + roasted**	19.4^d^	1.44^c^	8.6

^a^ΔE*ab colour interpretation: < 1 means not perceptible by the human eye, 1–2 means perceptible through close observation, 2–10 means perceptible at a glance, 11–49 means colours are more similar than opposite and 100 means colours are exact opposites.

Different superscript letters in a column indicate means that are significantly different.

Chroma indicates the degree in which a colour differs from the neutral colour of the same value whilst hue is defined as perception of colour of an object (i.e. red, orange, green, blue) [36]. Just like ΔE*ab trends, pre-treatments caused significant differences in chroma and hue (p > 0.05). Dry roasting resulted in higher chroma values than other processes whereas an opposite trend was observed for hue, with roasting decreasing hue values in both varieties. Colour differences are relevant as they affect consumer acceptability of products.

#### Colour of raw and pre-treated flour

Table 2 also shows ΔE*_ab_, chroma and hue values of flour made from red and black-eye varieties using different pre-treatment methods. Colour changes during roasting are ascribed to Maillard reactions occurring during roasting [35], influencing ΔE*_ab_, chroma and hue values of flour. The ΔE*_ab_ specifies differences between colour of control sample (raw) and pre-treated flour [36]. The ΔE*ab (0.14) of black-eye seeded raw and soaked flour shows that differences between flour were not perceptible by human eye. However, the ΔE*ab (1.2) of red seeded raw and soaked flour shows that differences between flour were perceptible through close observation. Exclusive roasting, combined soaking, and roasting processes are shown to have an effect on the colour of flour in both varieties such that colour differences from raw flour were perceptible at a glance. Even though soaking prior to roasting reduced ΔE*ab of flour of both varieties, differences in colour between exclusively roasted and combined soaked and roasted flour were still easily perceptible (ΔE*ab, 4.2–4.7). Chroma indicates the degree in which a colour differs from the neutral colour of the same value whilst hue is defined as perception of colour of an object (i.e. red, orange, green, blue) [36]. Just like ΔE*ab trends, pre-treatments caused significant differences in chroma and hue (p > 0.05). Dry roasting resulted in higher chroma values than other processes whereas an opposite trend was observed for hue, with roasting decreasing hue values in both varieties. Colour differences are relevant as they affect consumer acceptability of products.

#### Chemical composition of raw and pre-treated flour

Table 3 shows the chemical composition of flours from raw and pre-treated red and black-eye varieties. Pre-treating seeds decreased moisture content of flour, as also reported by Yusuf, Ayedun [37]. Moisture content of flour from red and black-eye seeds, i.e. 7.7 and 9.1%, respectively, was lower than the 11.3–11.6% reported by [34], but higher than the 4.3% reported by Yusuf, Ayedun [37].

**Table 3:** Chemical composition of differently processed flours of a red and a black-eye bambara groundnut variety.

Variety	Flour type	Moisture	Protein	Fat	Starch	Sucrose	Fructose
		g/100g DW					
Red	Raw	7.7 ± 0.0^cde^	17.1 ± 0.1^a^	7.0 ± 0.0^a^	50.4 ± 0.6^ab^	3.09 ± 0.38^ab^	0.31 ± 0.02^a^
	Soaked	7.7 ± 0.5^cde^	19.4 ± 0.3^b^	7.9 ± 0.1^ab^	48.3 ± 0.6^a^	2.24 ± 0.09^a^	0.30 ± 0.00^a^
	Dry roasted	6.1 ± 0.3^abc^	19.6 ± 0.1^b^	8.2 ± 0.0^b^	48.3 ± 0.6^a^	3.63 ± 0.12^bc^	0.32 ± 0.02^a^
	Soaked and roasted	5.0 ± 0.2^a^	19.5 ± 0.4^b^	8.5 ± 0.1^b^	47.8 ± 0.3^a^	2.35 ± 0.07^a^	0.23 ± 0.01^a^
Black-eye	Raw	9.1 ± 0.2^e^	15.6 ± 1.4^a^	7.6 ± 0.7^ab^	49.8 ± 0.4^ab^	4.42 ± 0.71^c^	0.23 ± 0.07^a^
	Soaked	8.0 ± 0.2^de^	17.1 ± 0.0^a^	8.3 ± 0.0^b^	52.0 ± 0.7^b^	1.94 ± 0.03^a^	0.26 ± 0.01^a^
	Dry roasted	6.9 ± 0.9^bcd^	17.0 ± 0.2^a^	8.6 ± 0.3^b^	51.4 ± 0.5^b^	4.65 ± 0.23^c^	0.32 ± 0.01^a^
	Soaked and roasted	5.9 ± 0.5^ab^	17.2 ± 0.0^a^	8.6 ± 0.0^b^	51.6 ± 1.3^b^	2.14 ± 0.08^a^	0.27 ± 0.01^a^

Different superscript letters in a column indicate means that are significantly different.

Protein content of flour from both varieties ranged from 15.6–19.6 g/100 g DW using a factor of 5.7 to convert nitrogen to protein. In previous studies 6.5 was used [23, 38]. The general use of 6.25 as N:P conversion factor is criticised because this disregards variations in non-protein nitrogen and variable percentages of nitrogen in individual proteins [38]. Yet the protein content of raw BG in this study is in line with the 17.8–19.7 g/100 g DW [34, 39], but lower than the 25 g/100 g reported by Brough, Azam-AIi [40]. The protein content of the flour of the red variety was significantly higher than that of the black-eye variety (p = 0.00), implying that choice of variety matters [35]. Moreover, pre-treatments significantly increased the protein content of flour of both varieties (p = 0.001).

However, as expected, no significant differences between pre-treatments were observed (p = 0.98) as thermal treatments do not change the amount of proteins.

The fat content of flour, which ranged from 7.0 to 8.6 g/100 g DW, agrees with the 6.0–10.4 g/100 g reported by Yusuf, Ayedun [37]. Fat content of the flour of the black-eye variety was significantly higher than that of the red variety (p = 0.027). As expected, no differences in fat content due to pre-treatments (p = 0.153) were observed. The low fat content in flour of raw seeds is ascribed to the larger amount of hulls as a result of poor dehulling efficiency, which lowered the fat content as hulls contain nearly no fat [41].

The starch content in flour from raw red and black-eye seeds, i.e. 50.4 and 49.8 g/100 g DW, respectively, is comparable to the 39–50% reported by Poulter [42], but lower than the 56.9–58.3 g/100 g DW reported by Deshpande, Sathe [43]. This dissimilarity is attributed to varietal differences [42], cultivation conditions [44] and analytical methods [15]. In comparison to other legumes, total starch content was comparable to the 41–44% in raw lentil, chickpea and red kidney beans [45], but higher than the 31–38% in raw cowpea [46].

The starch content of the red variety decreased significantly (p = 0.000) due to pre-treatment contrary to trends in pre-treated black-eye flour. The exhibited opposite behaviour in varieties is attributed to differences in the rate of starch hydrolysis due to presence of fibre, possible presence of natural enzyme inhibitors during analysis and inherent differences in starch structure and composition [47, 48]. Insignificant differences in starch content between pre-treatments (p = 0.792) were found, implying that differences observed in starch after pre-treatment were only due to varietal differences.

Fructose and sucrose contents ranged from 0.18 to 0.33 g/100 g DW and 1.9 to 5.0 g/100 g DW, respectively. Soaking decreased sucrose in both varieties, but the effect was more pronounced in the black-eye variety, which initially had a higher sucrose content. The current findings agree with Adebowale, Awolala [49], who also observed a decrease in carbohydrate content by soaking as well varietal differences. Longland, Barfoot [50] found that soaking affected both fructose and sucrose concentrations, but only sucrose in a significant manner. Significant differences in sucrose content between varieties (p = 0.021) were observed as opposed to insignificant effects on fructose (p = 0.145). Conversely, a significant difference was present between pre-treatments regarding sucrose and fructose contents (p > 0.05).

The ash content, which ranged from 2.5 to 3.2 g/100 g DW, agrees with the 2.8 g/100 g and 3.6–3.8 g/100 g DW reported by Abiodun and Adepeju [51] and Kaptso, Njintang [34], respectively. All pre-treated flours had a higher ash content than the flour from raw seeds, agreeing with Nti [35], who reported an increase in ash content after dehulling. The effect of pre-treatment on ash content was significantly higher in the red variety than in the black-eye variety (p = 0.000). Within pre-treatments, only the roasted and combined soaking and roasted flour had comparable ash contents. The flour from soaked seeds had a lower ash content.

### Effect of variety and pre-treatment on functional properties of flours

#### Thermal properties

Gelatinization temperatures and enthalpies of flours are presented in Table 4. According to Krueger, Knutson [52] and Tester [53], gelatinization temperatures are related to characteristics of the starch granule, such as the degree of crystallinity, starch composition and molecular structure of amylopectin. Gelatinization describes the irreversible disruption of molecular order within a starch granule when heated in excess water. The (T_0_) of raw flour, i.e. 77.1 and 76.9°C, was comparable to 76.8°C reported by Sirivongpaisal [54], but higher than the 67.4–71.2°C reported by Kaptso, Njintang [34].

**Table 4:** Gelatinization, LGC and pasting properties of differently processed flours of a red and a black-eye bambara groundnut variety.

Variety	Flour type	Onset T (°C)	Peak T(°C)	End T (°C)	ΔH (J/g)	LGC (%)	Peak	
							Time	Temperature
**Red**	**Raw**	77.1 ± 0.7^ab^	81.3 ± 0.7^ab^	85.7 ± 0.5^a^	4.8 ± 0.3^ab^	8	6.98 ± 0.04^c^	84.3 ± 0.5^a^
	**Soaked**	78.4 ± 0.6^bc^	82.6 ± 0.5^bc^	87.4 ± 0.3^abc^	4.5 ± 0.4^ab^	7	7.00 ± 0.00^c^	83.8 ± 0.4^a^
	**Dry roasted**	78.8 ± 0.1^c^	83.0 ± 0.0^c^	88.0 ± 0.2^bc^	5.2 ± 0.2^ab^	10	5.36 ± 0.15^a^	83.4 ± 0.5^a^
	**Soaked + roasted**	78.3 ± 0.1^bc^	82.8 ± 0.1^c^	88.8 ± 0.2^c^	5.6 ± 0.0^b^	6	5.25 ± 0.04^a^	83.8 ± 0.5^a^
**Black-eye**	**Raw**	76.9 ± 0.0^a^	81.0 ± 0.2^a^	85.7 ± 0.6^a^	4.1 ± 0.5^a^	6	6.98 ± 0.04^c^	84.0 ± 0.1^a^
	**Soaked**	77.1 ±0.1^ab^	81.6 ± 0.3^abc^	86.6 ± 0.3^ab^	4.1 ± 0.5^a^	6	7.00 ± 0.00^c^	83.6 ± 0.5^a^
	**Dry roasted**	78.6 ± 0.4^c^	82.5 ± 0.1^bc^	87.6 ± 0.5^abc^	5.1 ± 0.0^ab^	8	6.31 ± 0.54^b^	73.9 ± 16.8^a^
	**Soaked + roasted**	77.9 ± 0.1^abc^	81.8 ± 0.5^abc^	87.6 ± 0.8^abc^	5.0 ± 0.0^ab^	6	5.15 ± 0.04^a^	72.8 ± 17.9^a^

T_0_ is gelatinization onset temperature, T_p_ is gelatinization peak temperature, T_e_ is end of gelatinization temperature, and ΔH is enthalpy of gelatinization.

Different superscript letters in a column indicate means that are significantly different.

The substantial differences observed indicate differences in the starch granule, such as a more rigid granular structure in the red variety [55]. Lower onset (T_0_) and peak (T_p_) temperatures were obtained in the study as compared to cowpea (T_p_ = 86°C) [56]. Enthalpy of gelatinization (ΔH) of flour from raw seeds of the two varieties (4.1 and 4.8 J/g) was lower than the 6.0–9.7 J/g reported by Sirivongpaisal [54] and Kaptso, Njintang [34].

Pre-treatments increased gelatinization temperatures, indicating alterations in starch granule characteristics. In all cases, a significant effect of variety on gelatinization temperatures (p > 0.05) was observed. Basically, flour from the red variety had higher gelatinization temperatures than flour from the black-eye variety. Furthermore, a significant effect of pre-treatments on gelatinization temperatures (p > 0.05) was revealed. Pre-treatments resulted in similar values peak (T_p_) temperatures for both varieties, indicating that heat treatment hardly affected (T_p_). Pre-treated flour increased enthalpy of gelatinization (ΔH) and the same statistical trend as for gelatinisation temperatures was observed.

Gelatinization improves stiffening abilities of flour. In food applications, good gelatinization properties are important for thickening of soups and porridge [57]. During pre-treatment, lower gelatinization temperatures are therefore desirable, because of the lower energy requirements. However, differences in gelatinization temperature were fairly small, indicating a low variability in thermal properties of molecules, including starch granules that make up each variety [34]. Furthermore, differences between the onset and end temperature (T_e_-T_0_) were small, reducing the importance of this flour characteristic for food applications.

#### Least gelation concentration (LGC)

Table 4 shows the Least Gelation Concentration (LGC) of flour, which is defined as the lowest concentration of flour to form a self-supporting gel [8, 27]. Flour with a lower LGC has a greater gelling capacity. Gelation occurs due to protein denaturation, causing formation of hydrogen and ionic bonds stabilizing the gel and gelatinization of starch with other factors [26, 58]. Current LGC values, which varied from 6 to 10%, were comparable to the 8% reported by Eltayeb, Ali [29], but lower than the 12, 16 and 28% previously reported [7, 37, 59]. In comparison to other legumes, LGC of BG was higher than the 4% in groundnut [60], but lower than the 17% in cowpea [61].

Generally, black-eye flours had the least LGC values as compared to the red variety flours. This is credited to thee higher starch content of flours from the black-eye variety. Moreover, differences are also ascribed to variations in the relative ratio of protein and lipids and the interaction between these components [62]. In both varieties, roasting had a negative effect on LGC, but the effect was more pronounced in the red variety. The higher LGC in roasted samples is attributed to protein denaturation and dissociation because of the high roasting temperature (179°C). Protein subunits resulting from dissociation might not be favourable to form hydrogen and ionic bonds [63] required for gelation. Occurrence of Maillard reactions may have induced insoluble complexes that can result in reduced solubility [64].

Relative to simple sugars, Evageliou, Richardson [26] showed that the sucrose concentration has a marginal effect on LGC. In the current study, sucrose is the only factor that was changed by soaking, explaining why LGC of soaked flour does not differ much from raw flour. Gelation is important in creating texture in many food products, such as yogurt, processed meats and gelatin-based products [65]. All BG flours would do adequately in these products due to their low LGC, with roasted BG being the poorest performer.

#### Pasting properties

**Fig 4:**
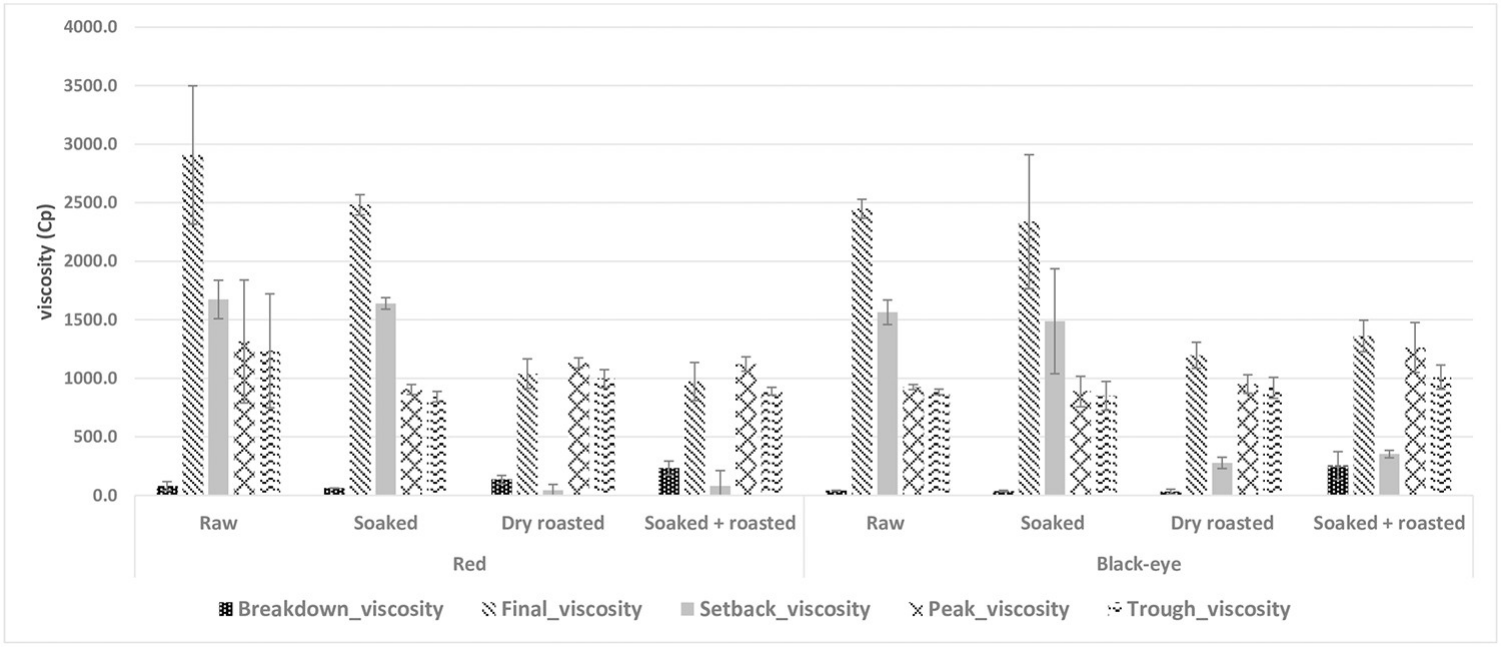
Viscosity properties of raw and pre-treated flours of a red and a black-eye bambara groundnut variety.

Pasting properties of raw and pre-treated bambara groundnut varieties are presented in Fig 4. Pasting includes the changes that occur after gelatinization upon further heating. These consist of further swelling of granules, leaching of molecular components such as amylose from the granules and eventual disruption of granules [66]. The properties of the swollen granules and the soluble materials leached from the granules control the viscosity parameters during pasting [67]. All pre-treatments decreased peak viscosity, except in combined soaking and roasting of the black-eye variety.

Peak viscosity indicates the water-binding capacity of the starch whereby the gelatinized starch reaches its maximum viscosity during heating in water [68]. Currently, insignificant differences were observed, implying that different treatments did not influence peak viscosity. High peak viscosity maybe suitable for products requiring high gel strength and elasticity. Moreover, a high peak viscosity indicates thermal stability of the flour, implying potential use in products requiring sterilization [69]. Trough viscosity measures the ability of the paste to withstand breakdown during cooling. It is the minimum viscosity value in the constant temperature phase of the Rapid Visco Analyser (RVA) pasting profile. Trough viscosity decreased in flours of the red variety, whereas an insignificant increase was recorded for the black-eye variety. High trough viscosity implies that the flour has the ability to remain undisrupted when subjected to a hold period of constant high temperature and mechanical stress by rapid and continuous mixing [68]. The breakdown viscosity in the pasting profile indicate the degree of disintegration of granules or paste stability. Less stable starch paste is commonly accompanied with a high value of breakdown. The higher the breakdown value, the lower the ability to withstand heating and shear stress during cooking. As low breakdown values are associated with stable gruels, soaked flour of both varieties resulted in stable pastes, whilst flours from the combined soaking and roasting pre-treatment resulted in unstable pastes for both varieties.

The final viscosity indicates the ability of the starch to form a viscous paste or gel after cooling. Final and setback viscosity decreased significantly in both varieties due to roasting, while an insignificant decrease was recorded in soaked flour of both varieties. Low final viscosity implies that flour will form a low viscous paste rather than a thick gel on cooking and cooling. Nutritionally, this means a high calorific density for a low volume [68]. The setback viscosity indicates the syneresis of starch upon the cooling of the cooked starch pastes [67]. High setback values are associated with a cohesive paste. Therefore, the higher the setback viscosity, the lower the retrogradation during cooling and the lower the starting of retrogredation for a product made from the flour.

Pasting temperature provides an indication of the minimum temperature required to cook a flour (Table 4). High pasting temperatures indicate a high water-binding capacity, gelatinization tendency, and lower swelling property of a starch-based flour due to a high degree of association between starch granules. Pasting temperature did not change much in the flours of the red variety, but decreased in both varieties. Peak time was decreased in dry roasted and combined soaked and roasted flour of both varieties, whilst soaking slightly increased it.

#### Emulsification properties

Emulsification capacity (EC) of raw and pre-treated flour ranged from 29 to 50% as shown in Table 5. EC and emulsion stability (ES) are indices to evaluate emulsifying properties of flours. EC measures the amount of oil that can be emulsified per unit of flour, whereas ES measures the ability of the emulsion to resist changes to its structure over a defined period [8]. EC is mainly determined by the solubility of the proteins, but insoluble proteins play a role as well, as do polysaccharides [70]. Protein can emulsify and stabilize an emulsion by decreasing the surface tension of the oil droplet and providing electrostatic repulsion on the surface of the oil droplet [71, 72]. EC of raw flours, i.e. 49.1 and 50.2%, was lower than the 65–69% of raw BG reported by Adebowale, Awolala [49]. In comparison to other legumes, EC was comparable to the 50% reported for pigeon pea [73].

**Table 5:** Functional properties of differently processed flours of a red and a black-eye bambara groundnut variety.

Variety	Pre-treatment method	EC (%)	ES (%)	FC (%)	FS (%)
**Red**	**Raw**	49.1 ± 2.7^d^	47.9 ± 1.0^cd^	10.8 ± 1.9^bcd^	79.0 ± 11.1^cd^
	**Soaked**	46.5 ± 1.4^cd^	46.5 ± 1.6^cd^	18.3 ± 1.9^ef^	79.1 ± 10.9^cd^
	**Dry roasted**	40.7 ± 0.7^b^	31.9 ± 0.7^b^	3.3 ± 3.1^a^	14.7 ± 4.8^a^
	**Soaked + roasted**	48.8 ± 3.0^d^	45.8 ± 3.4^cd^	7.5 ± 2.5^abc^	87.4 ± 3.8^d^
**Black-eye**	**Raw**	50.2 ± 1.4^d^	45.2 ± 1.1^c^	17.1 ± 1.9^def^	63.0 ± 8.4^bc^
	**Soaked**	48.7 ± 0.0^cd^	50.0 ± 0.6^d^	22.9 ± 1.4^f^	73.6 ± 5.7^bcd^
	**Dry roasted**	29.7 ± 2.8^a^	15.6 ± 2.2^a^	5.8 ± 2.9^ab^	0.0 ± 0.0^a^
	**Soaked + roasted**	43.4 ± 0.7^bc^	35.1 ± 0.8^b^	12.9 ± 3.1^cde^	57.2 ± 3.7^a^

Different superscript letters in a column indicate means that are significantly different.

ES of flours from raw seeds, i.e. 47.9 and 45.2%, were lower than the 70% reported for soaked BG flour by Adebowale and Lawal [59]. All pre-treatments significantly decreased EC and ES (p > 0.05), with roasting causing a greater decline, while soaking had little effect. Adebowale, Awolala [49] reported contrasting results on effects of soaking on EC of flour from black and cream BG varieties. Cold soaking decreased EC of cream variety flour, whereas an increase was observed in black variety flour. Currently, considering the varietal effect on EC, differences between combined soaking and roasting (46%), soaked flour (48%) and raw flour (50%) were small. Decreases in both EC and ES after roasting agree with Obatolu, Fasoyiro [74], who reported EC values ranging from 50.7% for raw to 20% for roasted beans and reported that processing resulted in significant reductions in EC for all treatments.

Good emulsification properties are important in many fat-containing food products, such as sausages, vegetable milk and milk-based products, as well as cake batters [72]. The negative effect of roasting on EC capacity and ES is ascribed to denaturation and dissociation of proteins and exposing hydrophobic regions, which increase surface tension [72]. Even though roasting reduced emulsification properties of BG flour, soaking prior to roasting seemed to mitigate the negative roasting effect. Soaking changes physical properties of flour, thereby decreasing the effect of heat treatments. The positive effect of soaking is ascribed to a change in thermal conductivity [75].

#### Foaming properties

Foams can be defined as a colloidal dispersions in which gas is the dispersed phase and liquid is the continuous phase [70]. Foaming in flours is induced by trapping air in water and stabilized by a decrease of surface tension by proteins [72]. Foaming capacity (FC) of raw and pre-treated flours ranged from 3.3 to 22.9% as shown in Table 5. FC of raw red and black-eye varieties, i.e. 10.8 and 17.1%, respectively, agrees with the 17–18% reported by Adebowale, Awolala [49]. In comparison to other legumes, FC was lower than for cowpea flour (40%) [76].

FC of the black-eye variety was significantly higher than of the red variety. FC was increased by soaking, whilst a decline was observed for roasting, agreeing with Obatolu, Fasoyiro [74] for roasted yam bean. Soaking prior to roasting counteracted the effects of roasting significantly in the black-eye variety, but not in the red variety. The effect of soaking on roasting is ascribed to the difference in heat transfer during roasting caused by soaking [77]. FS was comparable to the 80% after 1 h (with a FC of 70%) reported by Yusuf, Ayedun [37] and the 30% after 2 h [29]. FS of raw and soaked samples after 2 h was higher than for cowpea flour (71%) [76]. Roasting significantly decreased FS compared to soaked and raw flour. Soaking prior to roasting reduced the negative effect of roasting.

FC has been related to the amount of protein, with native protein having better foaming abilities than denatured protein [38]. This is due to the fact that proteins should be soluble in the aqueous phase, which happens most often at the isoelectric point [72]. Roasting is suggested to bring more hydrophobic areas of proteins to the surface, decreasing solubility. FC is important in food products such as cakes, soufflés and foams. Raw or soaked samples would be best for the production of this kind of products [72]. However, milder pre-treatment (70–80 °C) has also been reported to improve FC and FS by promoting denaturation to the extent that some hydrophobic regions of proteins come to the surface and resist reabsorption into the aqueous phase, making the protein film more dense [78].

#### Water and oil absorption

**Fig 5:**
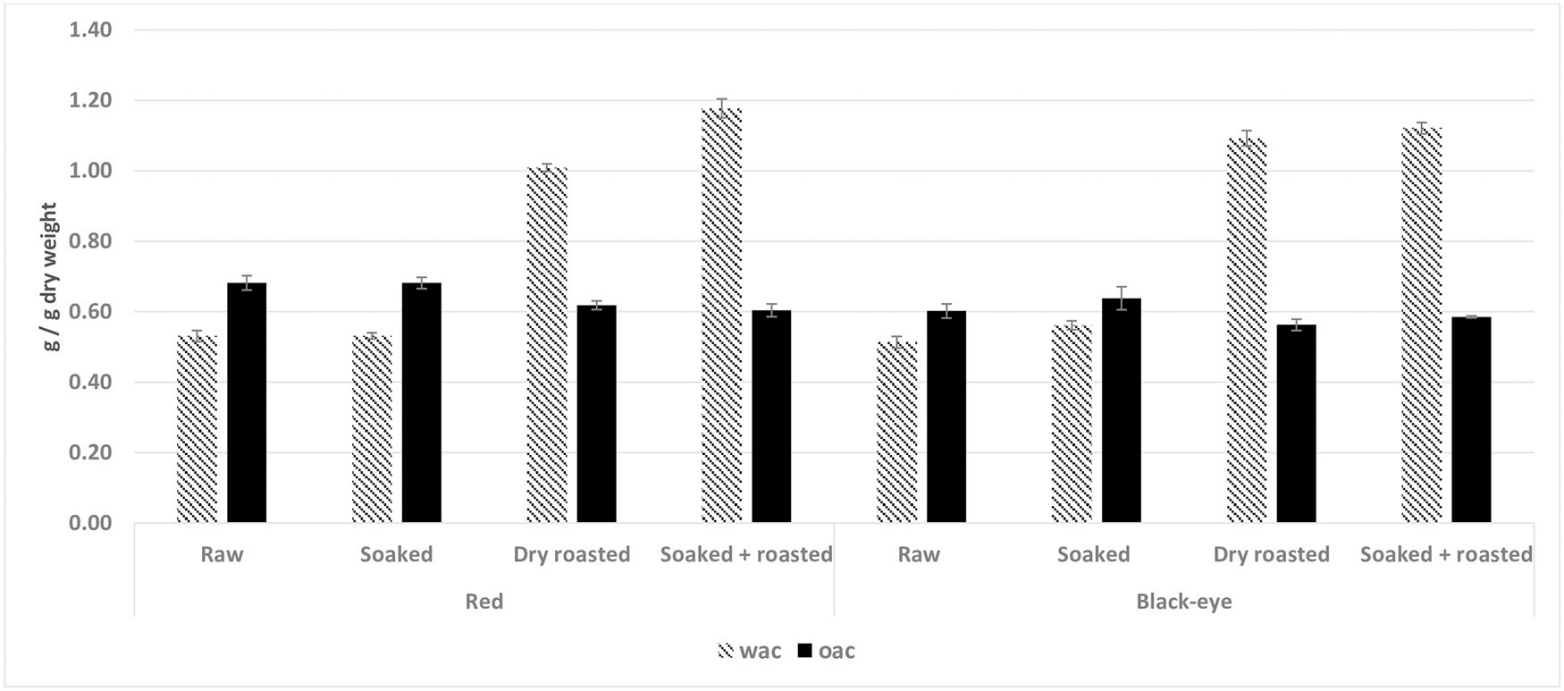
WAC and OAC properties of raw and pre-treated flours of a red and a black-eye bambara groundnut variety.

Water absorption capacity (WAC) of raw and pre-treated flours varied from 0.51 g/g to 1.12 g/g DW (Fig 5), which is lower than the 1.6 ml/g—2.8 ml/g DW previously reported [29, 31, 59]. In comparison to other legumes, WAC of BG flour was lower than the 2.1 g/g reported for roasted cowpea flour [76]. Soaking and dry roasting in the current study increased WAC, in line with Yusuf, Ayedun [37] for BG and [76] for cowpea. A high WAC in flour is attributed to denaturation and dissociation of proteins during roasting, which leave more polar binding sites than native protein [79]. A higher WAC enables bakers to add more water to doughs, improving handling and maintaining freshness in bread [80]. When considering the average WAC for both varieties, differences between raw and soaked flour are marginal (0.52 and 0.54 g/g, respectively), and the same trend appears for the dry roasted and combined soaking and roasting treatment (1.0 g/g and 1.1 g/g, respectively). Further, only pre-treatments significantly affected the WAC (p = 0.000).

Oil absorption capacity (OAC) of flours ranged from 0.56–0.68 g/g (Fig 5), which is lower than 1.3–2.0 ml/g DW reported by Diedericks and Jideani [31] and Adebowale, Afolabi [81]. In comparison to other legumes, the OAC was comparable to the 0.8 g/g reported for soaked cowpea flour [82], but lower than the 1.9 g/g for raw cowpea flour [76]. All pre-treatments increased the OAC in the red variety, but the opposite trend was observed in the black-eye variety. The positive effects of roasting on the OAC of legumes, which were confirmed by Yusuf, Ayedun [37], and Onimawo and Akpojovwo [83], are ascribed to emerging new binding sites that result from the dissociation and denaturation of protein during roasting. Oil absorption is important in oil-containing solid products, such as meat, sausages and donuts [72].

## Conclusion

All pre-treatment methods improved dehulling and milling efficiency of BG varieties cementing applicability in flour production. The decisive factor in selecting the preferred method for a specific food application has to focus on the sensorial, functional and nutritional properties of the flour. Colour was affected by roasting, such that roasted flour had a darker colour. Nutritionally, bambara groundnut flour compares with other legumes such as cowpea and chickpea. Varietal differences in chemical properties were minor, except for protein content and ash content, which were much higher in the red variety, making it considerably more nutritious. Consequently, pre-treatment is expected to have more effect on the functional properties of the red variety.

Differences in thermal properties due to varietal differences and pre-treatment methods were observed. The flours from the red variety had higher gelatinization temperatures as compared to the black-eye variety, whilst roasting caused a higher onset gelatinization temperature. Starch granule alterations are credited in the increase in gelatinization temperatures. Black-eye flours had lower LGC values than the red variety flours, which can be attributed to the higher starch content in the former.

Concerning food applications, the low emulsification and low foaming properties reported, suggests that BG flour is not suitable for food products that require a high percentage of porosity. Therefore, the solution to this will be blending with wheat to improve the properties. The high WAC of dry-roasted flour and combined soaked and roasted flour indicates usefulness of flour especially in food formulations involving dough due to its ability to absorb water and swell for improved consistency in food.

**Fig 6:**
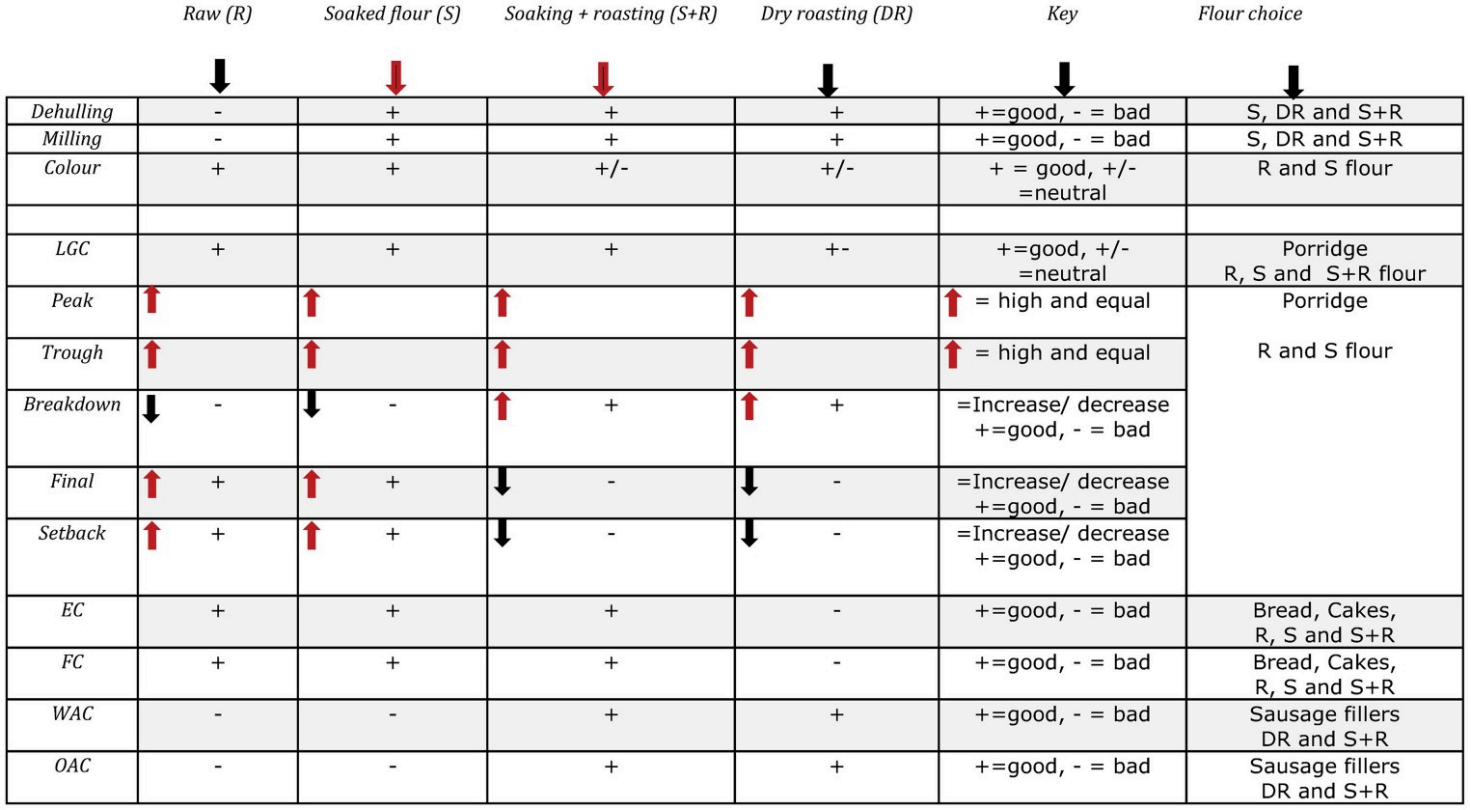
Potential applications of bambara groundnut flour in food products.

In conclusion, based on functional properties, both varieties are recommended for flour processing. No single processing method is optimal, but is determined by the intended application. Based on functional properties, the soaked and combined soaked and roasted flours are recommended for further research in product development and consumer acceptance of locally consumed food products such as porridge, soups, bread, cakes and fritters as illustrated in Fig 6. Nutrient enhancement of staple foods such as maize by blending with flour is also suggested as a way of increasing diversity while at the same time alleviating malnutrition problems faced by marginalised communities.
